# The new micro-kingdoms of eukaryotes

**DOI:** 10.1186/1741-7007-11-40

**Published:** 2013-04-15

**Authors:** Jan Pawlowski

**Affiliations:** 1 Department of Genetics and Evolution, University of Geneva, 30, Quai Ernest Ansermet, Sciences 3, CH-1211 Geneva, Switzerland

## 

Early metagenetic surveys of micro-eukaryotic diversity revealed numerous phylotypes that seemed unrelated to any described eukaryotic taxa. It has been proposed that some of them may represent novel kingdom-level taxonomic diversity in eukaryotes. However, detailed analysis of DNA sequences retrieved from environmental samples and assigned to these putative new kingdoms showed that most of them were undetected chimeras or incorrectly placed fast-evolving phylotypes [1]. Since then, the number of new eukaryotic lineages revealed by environmental surveys grew exponentially, but the question of how many of them deserve the highest taxonomic level has remained.

Over the past few years, reconstruction of deep eukaryote phylogeny focused on grouping various eukaryotic lineages into large monophyletic assemblages. Advances in phylogenomic studies led to the formation of four to seven supergroups (Figure 1). The classical multicellular kingdoms of animals and fungi are placed in the supergroup Opisthokonta, while green plants together with red algae form a supergroup of Archaeplastida. All other supergroups are composed of typically unicellular eukaryotes: Amoebozoa comprise the lobose amoebae and slime molds, Stramenopiles include among others diatoms, kelps, and oomycetes (previously classified as fungi), Alveolata are composed of ciliates, dinoflagellates and parasitic apicomplexans (among which the causative agents of malaria and toxoplasmosis), Rhizaria are dominated by amoeboid protists, such as foraminifera, radiolarians or filose amoebae, and heterotrophic flagellates with filose pseudopodia, and Excavata group together euglenozoans, heterolobosean amoebae and some amitochondriate parasitic phyla (such as diplomonads and parabasalids). However, with an increasing amount of phylogenetic and metagenetic data available for larger taxon sampling of eukaryote diversity, there was growing evidence that not all lineages could be placed inside the established supergroups. More than ten lineages were considered as of uncertain placement and left as *incertae sedis *in the recently revised eukaryote classification [2].

**Figure 1:**
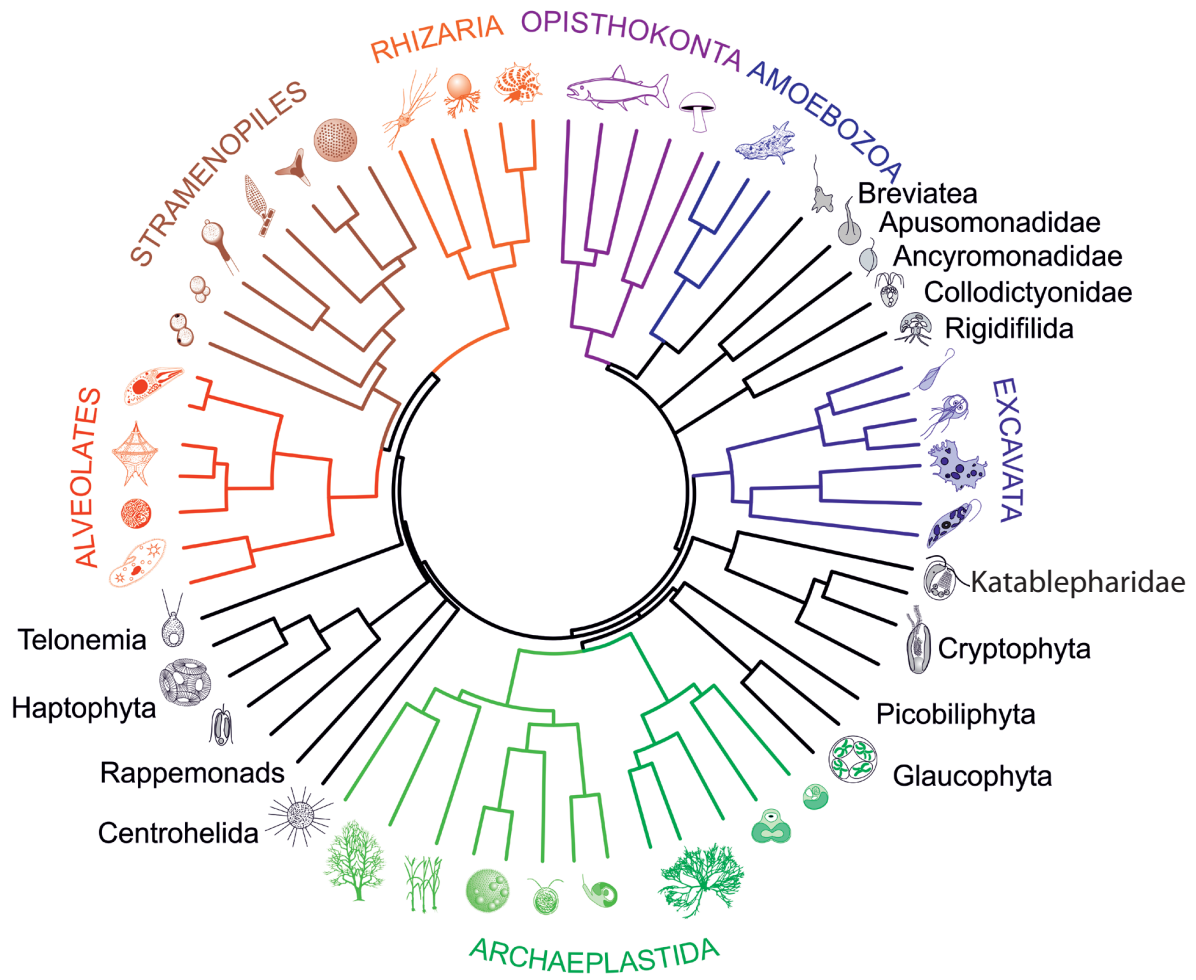
**Deep phylogeny of eukaryotes showing the position of small eukaryotic lineages that branch outside the seven supergroups (modified after Burki *et al***. [12]; drawings S Chraiti).

What are these hypothetical novel eukaryotic micro-kingdoms? First of all, many of them are not really new. For example, the genus *Collodictyon *was described 150 years ago but the diversity and evolutionary importance of Collodictyonidae have been investigated only recently [3]. The unclassified eukaryotic lineages represent a large variety of forms and modes of life, from free-swimming or gliding biflagellated cells to amoeboflagellates or filopodia-bearing amoeboid cells. Some lineages have cells covered with mineralized scales or spicules (Haptophyta, Centrohelida) or organic thecae (Apusomonadidae, Rigidifilida). Few lineages are phototrophs and possess chloroplasts acquired as a result of the secondary symbiosis (Cryptophyta, some Haptophyta). Most of them are mixotrophs or heterotrophs, often bacterivorous, living in marine or freshwater environments. Only one lineage (Breviatea) is amitochondriate and lives in anaerobic conditions, but interestingly no group comprises parasitic species. With the exception of Haptophytes and Cryptophytes, all other lineages are represented by very few described species, although their environmental diversity may be quite large. For example, the genus *Telonema*, represented by only two described morphospecies, has seen its diversity explode with more than 20 phylotypes revealed by environmental study [4].

Metagenetic studies played a key role in discovery and assessment of the diversity of these putative micro-kingdoms. Two lineages (Picobiliphytes, Rappemonads) are known exclusively from environmental sequence data [5,6]. Very little is known about their morphology and cell characteristics and in some cases even the features that seemed to be well established, like the presence of chloroplasts in Picobiliphytes, have been questioned based on a single-cell genomic study [7]. Compared to some other planktonic groups revealed by metagenetics, such as marine stramenopiles (MAST) or marine alveolates (MALV) [8], the environmental diversity of novel lineages seems relatively modest, though some of them have been found to be quite diverse when specifically targeted [9].

The phylogenetic position of these lineages remained an unresolved conundrum, in spite of genomic and/or transcriptomic data available for most of the groups that include cultivable species. A new supergroup, called Hacrobia or CCTH has been created for some of these orphan lineages [10,11], but later analyses did not support this hypothetical grouping [12]. An up-to-date phylogenomic tree (Figure 1) shows 13 independent lineages branching in three paraphyletic assemblages placed at the base of SAR assemblage grouping Stramenopiles, Alveolata and Rhizaria (Telonema, Haptophytes, Rappemonads, Centrohelids), next to Archaeplastida (Glaucophytes, Picobiliphytes, Cryptophytes, Katablepharids), and close to Amoebozoa (Breviatea, Collodictyon, Rigidifilida, Apusomonads, and Ancyromonads). Phylogenetic relations between these lineages and the adjacent supergroups are not well established. Some of them, like Breviatea and Glaucophyta are included in Amoebozoa and Archaeplastida, respectively. However, support for these groupings is usually weak and there is no general consensus about their classification.

One of the most striking characteristics of these orphan lineages and the reason why it is so difficult to place them in the eukaryotic tree is their extreme genetic divergence. High genetic distances separating them from other supergroups suggest that they represent the deepest eukaryotic lineages. Identifying and characterizing these deep lineages is essential for understanding the early evolution of eukaryotes. However, their number and diversity is difficult to assess. It has been assumed that many new deep lineages will be revealed by next-generation sequencing surveys of environmental diversity but their identification using short and standardized sequence tags [13] might not be straightforward. The single-cell genomic approach [7] may represent a much more efficient way to unveil the diversity of these unique lineages and establish their phylogenetic position.

Our view on the dawn of eukaryote evolution has profoundly changed as a result of phylogenetic and metagenetic studies. The traditional oversimplified classification of unicellular eukaryotes based on their mode of locomotion or capacity to photosynthesize has been replaced by a phylogenetically robust system of large, monophyletic eukaryotic supergroups that originated more than 1 billion years ago. Still, relatively little is known about the radiation of numerous small lineages that preceded the divergence of these supergroups. The challenge of future eukaryotic research is to describe the vastness of this radiation and to determine the ecological and evolutionary importance of the new micro-kingdoms.

## Note

This article is part of the BMC Biology tenth anniversary series. Other articles in this series can be found at http://www.biomedcentral.com/bmcbiol/series/tenthanniversary.

## References

[B1] Berney C Fahrni J Pawlowski J How many novel eukaryotic "kingdoms"? Pitfalls and limitations of environmental DNA surveys BMC Biol 2004 2 13 10.1186/1741-7007-2-13 15176975 PMC428588

[B2] Adl SM Simpson AG Lane CE Lukeš J Bass D Bowser SS Brown MW Burki F Dunthorn M Hampl V Heiss A Hoppenrath M Lara E Le Gall L Lynn DH McManus H Mitchell EA Mozley-Stanridge SE Parfrey LW Pawlowski J Rueckert S Shadwick L Schoch CL Smirnov A Spiegel FW The revised classification of eukaryotes J Eukaryot Microbiol 2012 59 429 493 10.1111/j.1550-7408.2012.00644.x 23020233 PMC3483872

[B3] Zhao S Burki F Bråte J Keeling PJ Klaveness D Shalchian-Tabrizi K Collodictyon--an ancient lineage in the tree of eukaryotes Mol Biol Evol 2012 29 1557 1568 10.1093/molbev/mss001 22319147 PMC3351787

[B4] Bråte J Klaveness D Rygh T Jakobsen KS Shalchian-Tabrizi K Telonemia-specific environmental 18S rDNA PCR reveals unknown diversity and multiple marine-freshwater colonizations BMC Microbiol 2010 10 168 10.1186/1471-2180-10-168 20534135 PMC2891722

[B5] Not F Valentin K Romari K Lovejoy C Massana R Töbe K Vaulot D Medlin LK Picobiliphytes: a marine picoplanktonic algal group with unknown affinities to other eukaryotes Science 2007 315 253 255 10.1126/science.1136264 17218530

[B6] Kim E Harrison JW Sudek S Jones MD Wilcox HM Richards TA Worden AZ Archibald JM Newly identified and diverse plastid-bearing branch on the eukaryotic tree of life Proc Natl Acad Sci USA 2011 108 1496 1500 10.1073/pnas.1013337108 21205890 PMC3029697

[B7] Yoon HS Price DC Stepanauskas R Rajah VD Sieracki ME Wilson WH Yang EC Duffy S Bhattacharya D Single-cell genomics reveals organismal interactions in uncultivated marine protists Science 2011 332 714 717 10.1126/science.1203163 21551060

[B8] Logares R Audic S Santini S Pernice MC de Vargas C Massana R Diversity patterns and activity of uncultured marine heterotrophic flagellates unveiled with pyrosequencing ISME J 2012 6 1823 1833 10.1038/ismej.2012.36 22534609 PMC3446805

[B9] Liu H Probert I Uitz J Claustre H Aris-Brosou S Frada M Not F de Vargas C Extreme diversity in noncalcifying haptophytes explains a major pigment paradox in open oceans Proc Natl Acad Sci USA 2009 106 12803 12808 10.1073/pnas.0905841106 19622724 PMC2722306

[B10] Okamoto N Chantangsi C Horák A Leander BS Keeling PJ Molecular phylogeny and description of the novel katablepharid Roombia truncata gen. et sp. nov., and establishment of the Hacrobia taxon nov PLoS One 2009 4 e7080 10.1371/journal.pone.0007080 19759916 PMC2741603

[B11] Burki F Inagaki Y Brate J Archibald JM Keeling PJ Cavalier-Smith T Horak A Sakaguchi M Hashimoto T Klaveness D Jakobsen KS Pawlowski J Shalchian-Tabrizi K Early evolution of eukaryotes: two enigmatic heterotrophic groups are related to photosynthetic chromalveolates Genome Biol Evol 2009 1 231 238 20333193 10.1093/gbe/evp022 PMC2817417

[B12] Burki F Okamoto N Pombert JF Keeling PJ The evolutionary history of haptophytes and cryptophytes: phylogenomic evidence for separate origins Proc Biol Sci 2012 279 2246 2254 10.1098/rspb.2011.2301 22298847 PMC3321700

[B13] Pawlowski J Audic S Adl S Bass D Belbahri L Berney C Bowser SS Cepicka I Decelle J Dunthorn M Fiore-Donno AM Gile GH Holzmann M Jahn R Jirků M Keeling PJ Kostka M Kudryavtsev A Lara E Lukeš J Mann DG Mitchell EA Nitsche F Romeralo M Saunders GW Simpson AG Smirnov AV Spouge JL Stern RF Stoeck T CBOL Protist Working Group: Barcoding Eukaryotic Richness beyond the Animal, Plant, and Fungal Kingdoms PLoS Biol 2012 10 e1001419 10.1371/journal.pbio.1001419 23139639 PMC3491025

